# Theoretical Investigation into Polymorphic Transformation between β-HMX and δ-HMX by Finite Temperature String

**DOI:** 10.3390/molecules29204819

**Published:** 2024-10-11

**Authors:** Xiumei Jia, Zhendong Xin, Yizheng Fu, Hongji Duan

**Affiliations:** 1 School of Innovation and Entrepreneurship, North University of China, Taiyuan 030051, China; 2 Department of Admission and Employment, North University of China, Taiyuan 030051, China; xzhend@nuc.edu.cn; 3 School of Materials Science and Engineering, North University of China, Taiyuan 030051, China; fuyizheng@nuc.edu.cn (Y.F.); duhongji1983@163.com (H.D.)

**Keywords:** polymorphic transformation between β-HMX and δ-HMX, molecular crystal, finite temperature string, order parameters, temperature effect on nucleation, Markovian milestoning with Voronoi tessellations, K-means clustering

## Abstract

Polymorphic transformation is important in chemical industries, in particular, in those involving explosive molecular crystals. However, due to simulating challenges in the rare event method and collective variables, understanding the transformation mechanism of molecular crystals with a complex structure at the molecular level is poor. In this work, with the constructed order parameters (OPs) and K-means clustering algorithm, the potential of mean force (PMF) along the minimum free-energy path connecting *β*-HMX and *δ*-HMX was calculated by the finite temperature string method in the collective variables (SMCV), the free-energy profile and nucleation kinetics were obtained by Markovian milestoning with Voronoi tessellations, and the temperature effect on nucleation was also clarified. The barriers of transformation were affected by the finite-size effects. The configuration with the lower potential barrier in the PMF corresponded to the critical nucleus. The time and free-energy barrier of the polymorphic transformation were reduced as the temperature increased, which was explained by the pre-exponential factor and nucleation rate. Thus, the polymorphic transformation of HMX could be controlled by the temperatures, as is consistent with previous experimental results. Finally, the HMX polymorph dependency of the impact sensitivity was discussed. This work provides an effective way to reveal the polymorphic transformation of the molecular crystal with a cyclic molecular structure, and further to prepare the desired explosive by controlling the transformation temperature.

## 1. Introduction

Polymorphism is a common phenomenon, where a system with specific molecular components can stack into different lattices, accompanied by different crystal properties [1,2,3,4,5,6,7]. At the molecular level, understanding the mechanism of polymorphism transformation is of great importance for clarifying the structure–property relationship of the polymorph materials [8,9] in energetic materials [10], nonlinear optical materials [11], pharmaceuticals [12], etc., so as to provide microkinetic [13] and thermodynamic [14] information for the desired polymorph during the transformation process in industrial production. In particular, since polymorphism can seriously affect the stability and detonation performance of the explosive [15], it is very important to reveal the polymorphic transformation mechanism and obtain the desired single-crystal form with high-energetic and insensitive properties [16,17,18,19].

HMX is one of the most important energetic materials, and it has been widely used in various mixed explosives and propellants. It has four kinds of polymorphism, i.e., *α*, *β*, *γ*, and *δ* forms. Under atmospheric pressure and room temperature, *β*-HMX exists stably with the highest energy. Therefore, *β*-HMX has been used in most of the explosive formulations. However, within the range of 165~210 °C, or under the mechanical impact, thermal stimulation, or shock waves, *β*-HMX can be transformed into the highly sensitive *δ*-HMX [20]. Studying the *β* → *δ* or *δ* → *β* polymorphic transformation can deepen understanding of the sensitivity, detonation process, and storage safety of HMX. Smilowitz et al. investigated the kinetics of the *β* → *δ* transformation [21,22] and discussed the nucleation mechanisms of the *δ*-form, and found that the kinetics were controlled by the melting of *δ*-HMX. The *β* → *δ* transformation free energy was calculated to be about 200 kJ/mol, consistent with the experimental result from Brill [23]. Weese [24] studied the *β* → *δ* kinetics of HMX by differential scanning calorimetry and confirmed that the *β* → *δ* polymorphic transformation was a multi-step process. Furthermore, the polymorphic transformation of HMX is greatly influenced by the temperature. Cady et al. [20] have found that the degree of the *β* → *δ* transformation depends on the final temperature, and when the temperature decreases (lower than 170 °C), the direction of transformation will be reversed, i.e., from *δ*-HMX to *β*-HMX. Smilowitz et al. [22] have also observed that during the cooling process, the reverse process of the *β* → *δ* transformation of HMX will occur, and the transformation rate of the reverse process can be controlled by the cooling rate. The *β* → *δ* kinetic process was described by four reaction rate constants involving the temperature [25].

Today, although many transformation mechanisms for molecular crystals have been confirmed by experiments [5,6,7,13,26,27] and theories [28,29,30], such as nucleation [31], concerted, transformations [32], surface-mediated [33], etc., it is still controversial how the original forms are broken and the molecules aggregate into the new crystal during the polymorphism process [34,35,36,37,38]. Undoubtedly, it is very difficult to reveal it in an experiment. As for the molecular dynamics (MD) simulations, the conventional method is difficult to implement since the timescale for them is many orders of magnitude lower than that of real occurrence [39,40]. Firstly, the nucleation in the polymorphism transformation process is a rare event [41,42], which is difficult to describe by using the standard MD method [43]. Secondly, due to the similar crystal structure of the polymorphic forms [44], it is very difficult to find collective variables that can be used to describe their reaction coordinate [41,45].

Recently, many rare event methods have been developed to describe the path of the phase transition via the free-energy landscape [46,47,48,49,50,51,52,53]. Finite temperature string (FTS) is one of the important path-based rare event methods for the reparameterization of each image in a path [52,54,55,56], and a minimum free-energy path (MFEP) [57] could be obtained with the string method in collective variables (SMCV) [57,58]. Indeed, FTS has been used to explore the MFEPs for the conformational transformation of biomacromolecules [59,60,61,62,63,64,65,66].

To describe the motion of molecules by the collective variable [45] during the phase transitions, the order parameter (OP) that contains all the variables with the molecular structures was used [67]. Although OPs have been used to describe the nucleation of molecular crystals at the molecular level [41,66,68,69,70,71], they were rarely applied in the description of the nucleation of the polymorphic transformation of crystals with a ring-shaped molecular structure [70,71].

Sampling is a challenge for the FTS method with the collective variable OP in exploring the phase transition process for complex systems [39,55,72], and improper sampling can not only lead to string roughness, but also result in dimensionality explosion. K-means clustering is an algorithm by which a data set containing *n*-dimensional vectors are clustered and divided into *k* sub-clusters by the iterations [73], and it has become an important means of enhancing sampling in machine learning [74,75,76].

In this work, a K-means clustering algorithm for the constructed OPs of HMX was used to optimize the string, so that an MFEP connecting *β*-HMX and *δ*-HMX was found quickly by the SMCV method, and the potential of mean force (PMF) was obtained. Thus, the free-energy profile was constructed by Markovian milestoning with Voronoi tessellations [55,77,78,79]. The influences of the temperature on nucleation were also investigated. This work expands the application of FTS to the polymorphic transformation of explosive molecular crystals with a complex ring-like molecular structure, and it is useful in screening high-energetic and insensitive explosives in industrial production.

## 2. Results and Discussion

### 2.1. Peaks in Pair Distribution Function of β-HMX and δ-HMX

**Figure 1:**
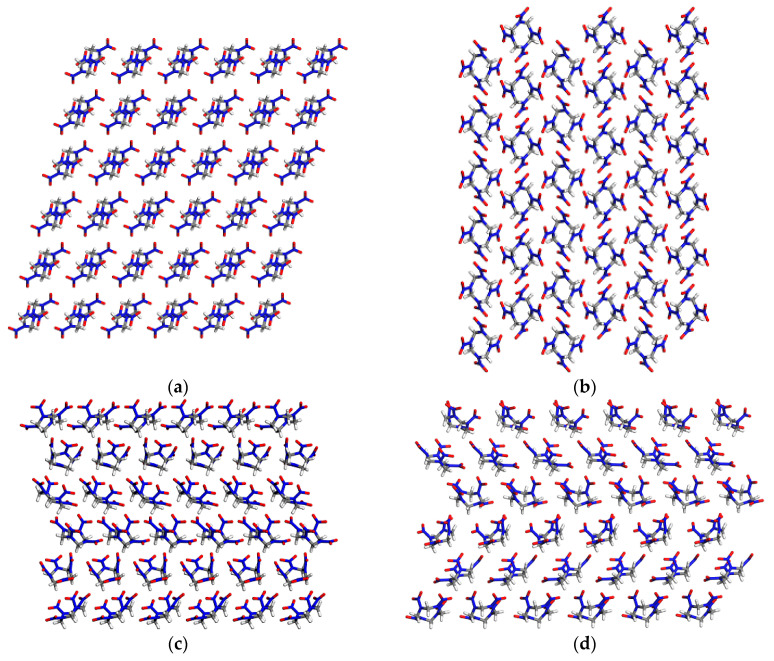
*β*-HMX crystal structure with the size of 6 × 6 × 6 (216 molecules) from the **b**-axis view (**a**) *β*-HMX (a–c plane) and (**b**) *β*-HMX (b–c plane), and the corresponding *δ*-HMX crystal structure in (**c**) *δ*-HMX (a–c plane) and (**d**) *δ*-HMX (b–c plane). Red, blue, gray, and white represent O, N, C, and H atoms, respectively.

**Figure 2:**
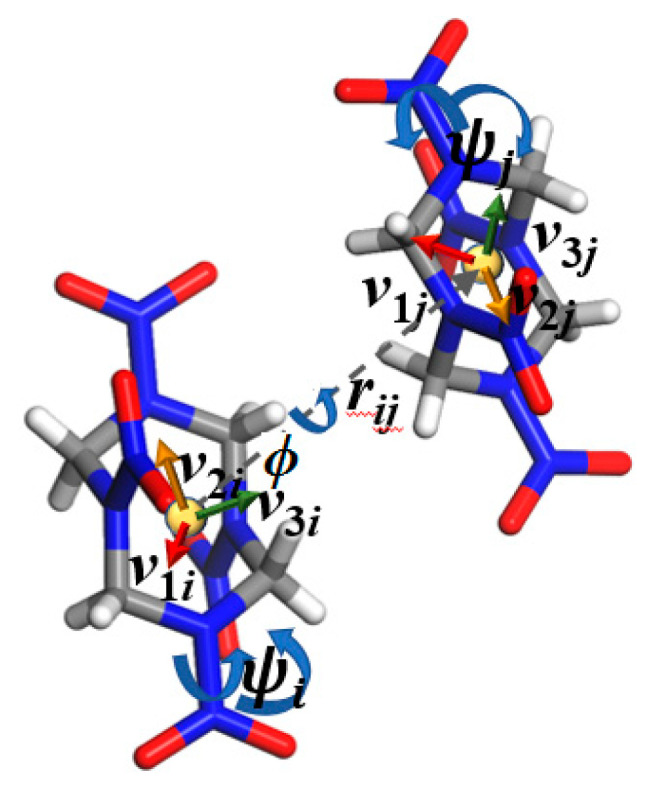
An illustration of the OP construction for HMX. The vector ***r*** joins the center of mass of the two HM molecules (${\hat{r}}_{ij}$). The direction of the axis passing through the center of the six-membered ring and perpendicular to the plane formed by the three C atoms or three N atoms on the ring is used as an approximate measure of the absolute orientation (*q_i_* or *q_j_* for molecule *i* or *j*). The bond orientation $\phi_{\hat{r}}$ defined as the projection of ${\hat{r}}_{ij}$ onto *q_i_* or *q_j_*, and the relative orientation $\phi_{q}$ that shows the rotates of *n_i_* onto *n_j_* (n = 1, 2, and 3).

Based on the experimental results [80] (see Table S1), *β*-HMX (P2_1_/c, Monoclinic) and *δ*-HMX (P6_1_, cubic) crystal structures were constructed with the size of 6 × 6 × 6 (see Figure 1). According to the OP construction strategy [41], the OPs of HMX were built (see Figure 2). The peaks in the pair distribution functions of *β*-HMX and *δ*-HMX are given in Table S2 (480 K with a cutoff of 10.0 Å), and the corresponding average peak locations and concentration parameters are collected in Table S3. In most cases, there are significant differences in the corresponding average peak positions between two polymorphs, showing that the constructed OPs in this work are suitable for distinguishing two crystal forms. Therefore, the bond orientation OPs and relative orientation OPs were chosen to describe the transformation of two HMX crystal forms.

### 2.2. Convergence of FTS and K-Means Clustering

**Figure 3:**
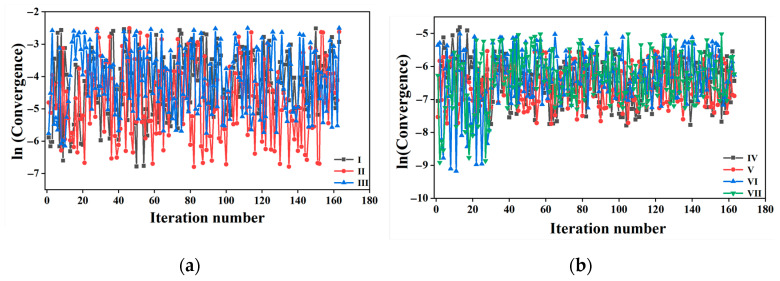
Convergence of collective variables during the evolution of the string. (**a**): (I), (II), and (III) orderly correspond to $\theta_{C}^{d}$, $\theta_{C}^{b}$, and $\theta_{C}^{r}$ OPs as the collective variables for K-means clustering sampling, respectively. (**b**): (IV), (V), (VI), and (VII) orderly correspond to $\theta_{C}^{db}$ and $\theta_{C}^{dr}$ with K-means clustering, $\theta_{C}^{dr}$ and $\theta_{C}^{db}$ with the average-based sampling, respectively.

In the previously accepted FTS method, sampling is often average-based. In this work, the average-based sampling was first adopted for the divided 20 parallel independent spaces along the path, and the values of the peaks are shown in Table S4. Most of the values of 1/*σ*^2^ are lower and the peak values are uncertain, indicating that the sampling is disperse and uncertain. Thus, there is always sampling in one space involving a certain replica covering the adjacent spaces involving the other regions, leading to evolution confusion. For example, for the $\theta_{C}^{db}$ and $\theta_{C}^{dr}$ Ops as the collective variables, after more than 150 iterations, the string was not converged (see Figure 3, average-based sampling).

In order to accelerate convergence, for all the samples of each of the replicas from the simulation times of 3 × 10 ns, the density and weight of each sample were calculated, and a K-means clustering algorithm based on the Euclidean distance and sample weight was adopted. The results show that, similar to a recent investigation [70], most of the partition coefficient (PC) values are greater than 0.75, and the partition entropies (PEs) are less than 0.45 (see Table S5). These results show that the K-means clustering algorithm was available to the samples. Indeed, the average smoothing score is no more than 50.0, while it is more than 120.0 for the average-based sampling. As a result, a smooth initial string was formed by the K-means clustering.

Figure 3a gives the convergence of the $\theta_{C}^{d}$, $\theta_{C}^{b}$, and $\theta_{C}^{r}$ OPs as the collective variables for the K-means clustering sampling. After more than 160 iterations, they still do not converge. Meanwhile, for $\theta_{C}^{db}$ and $\theta_{C}^{dr}$, the strings were converged after no more than 30 and 35 iterations, respectively, as shown in Figure 3b. This confirms the effectiveness and advantage of the K-means clustering $\theta_{C}^{db}$ and $\theta_{C}^{dr}$ as the collective variables for FTS. Therefore, K-means clustering is more available for sampling than average-based sampling, which is in agreement with previous investigations [81,82,83].

### 2.3. Minimum Free-Energy Path

The PMF is the potential energy obtained by integrating the average force of the configuration ensemble, and it indicates the maximum likelihood path of the MFEP [35,45,57,79,84,85].

**Figure 4:**
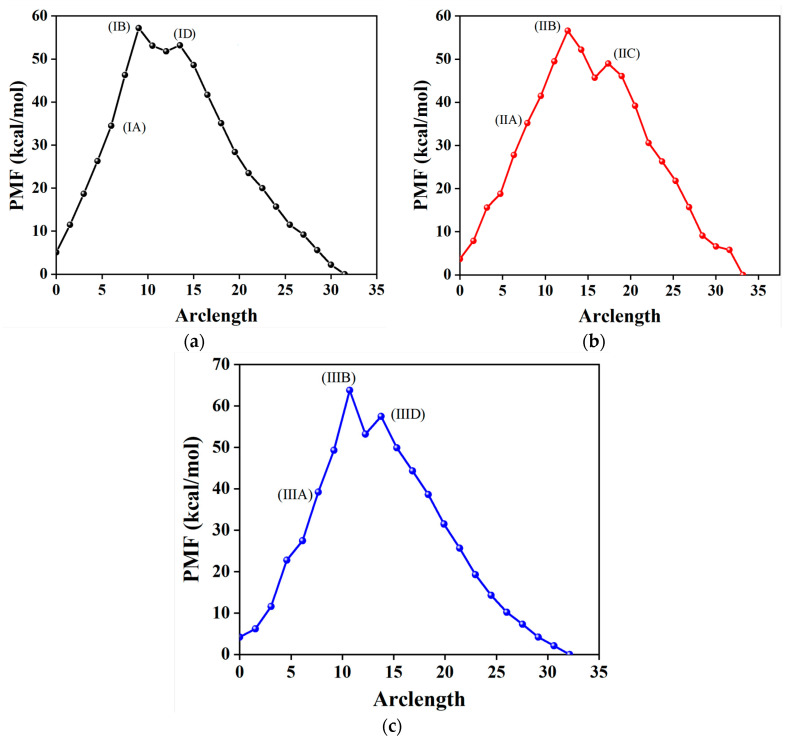
PMF as a function of the arclength along the FTS path. The initial point at arclength zero is *δ*-HMX, and the end point is *β*-HMX. (I), (II), and (III) are orderly the FTS path from $\theta_{C}^{db}$ and $\theta_{C}^{dr}$ with K-means clustering, $\theta_{C}^{dr}$ without K-means clustering, respectively. (**a**,**b**) mean the PMF curves corresponding to the $\theta_{C}^{db}$ and $\theta_{C}^{dr}$ with the K-means clustering sampling, and (**c**) is the PMF curve involving $\theta_{C}^{dr}$ from the average-based sampling.

Figure 4 shows three PMF curves. Among them, (I) and (II) mean the PMF curves corresponding to the $\theta_{C}^{db}$ and $\theta_{C}^{dr}$ with the K-means clustering sampling, and (III) is the PMF curve involving $\theta_{C}^{dr}$ from the average-based sampling. For each curve, there is always one metastable intermediate with a notable transition state (left) and an unapparent one (right). Interestingly, the difference in the PMF values between *δ*-HMX and the left transition state is close to each other, with values of about 52.0 kcal/mol. This result is larger than that of the experimental values of 200.0 kJ/mol [22,23]. It is noted that the values of the PMF are in general overestimated compared to the true value [70]. The difference between *β*-HMX and the right transition state is also close to each other. This shows that the potential barrier of the polymorphic transformation, whether from $\theta_{C}^{db}$ or $\theta_{C}^{dr}$, is the same and it is independent from the type of OP, which is in agreement with the previous investigation [70]. However, the difference in the PMF values between *δ*-HMX/*β*-HMX and the transition state from (III) with the average-based sampling is obviously larger than that from (I) and (II), showing that the selection of the sampling algorithm model, i.e., with or without K-means sampling, affects the accuracy of the PMF values.

**Figure 5:**
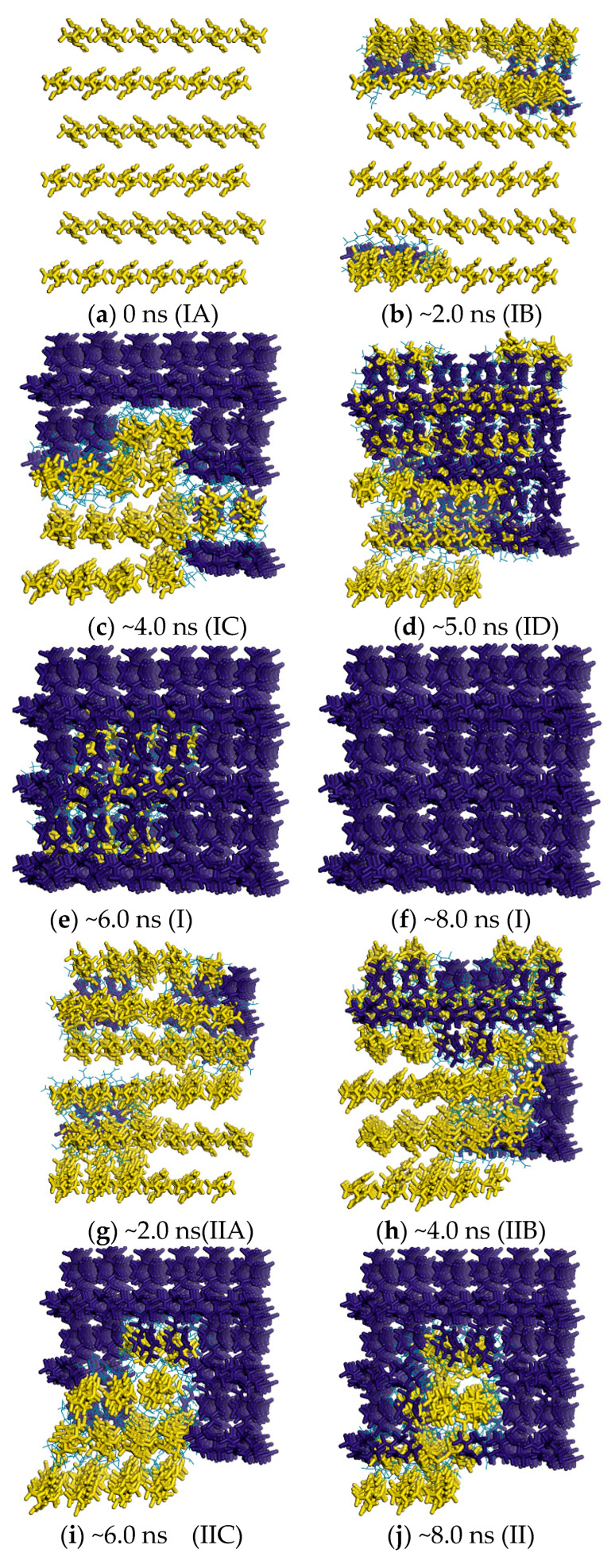

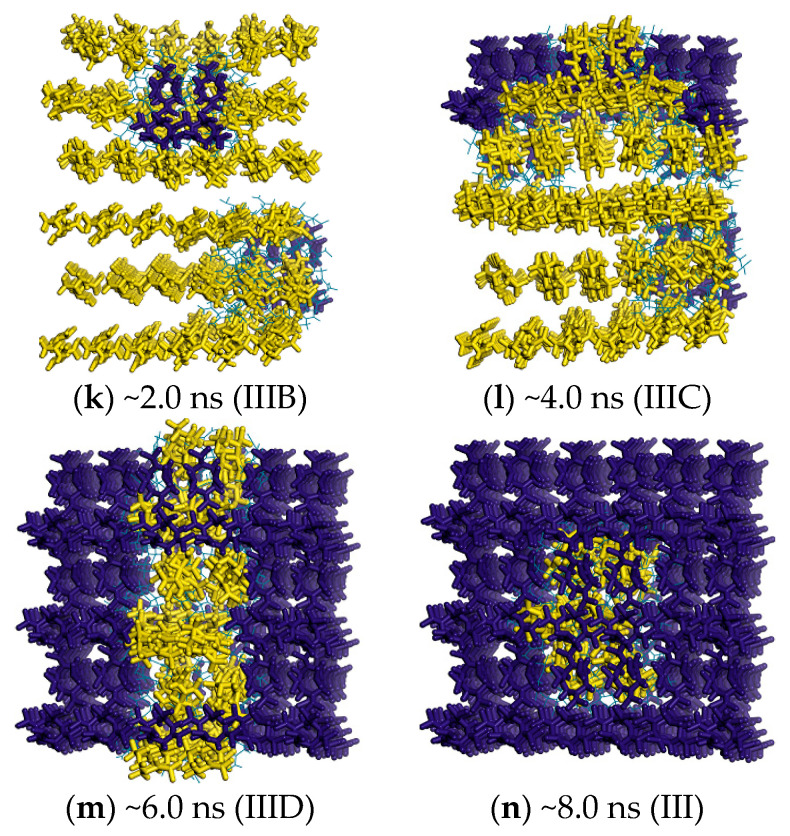
Changes in the local order parameters on the FTS path and times (“~” represents “approximately”); 0 ns (IA) ~2.0 ns (IB) ~4.0 ns (IC) ~5.0 ns (ID) ~6.0 ns ~8.0 ns (I) $\theta_{C}^{db}$ with K-means clustering (IIA) ~2.0 ns (IIB) ~4.0 ns (IIC) ~6.0 ns ~8.0 ns (II) $\theta_{C}^{dr}$ with K-means clustering (IIIA) ~2.0 ns (IIIB) ~4.0 ns (IIIC) ~6.0 ns (IIID) ~8.0 ns (III) $\theta_{C}^{dr}$ without K-means clustering.

To further reveal the mechanism of the polymorphic transformation between *δ*-HMX and *β*-HMX, the local OPs are shown for the key snapshots (see Figure 5). The molecules existing in the *β*-HMX form are labeled in yellow with the values of $\theta_{C}^{db}$ > ~250 or $\theta_{C}^{dr}$ > ~280, and the dark blue molecules are *δ*-HMX with the values of $\theta_{C}^{db}$ < ~180 or $\theta_{C}^{dr}$ < ~220, whereas the wireframe molecules in baby blue form are the interface between *δ*-HMX and *β*-HMX with the OP range of 180~250 for $\theta_{C}^{db}$ or 220~280 for $\theta_{C}^{dr}$. From left to right, the number of molecules with a high OP value decreases gradually, and the polymorphic transformation process moves from *β*-HMX to *δ*-HMX. Thus, by utilizing the $\theta_{C}^{db}$ and $\theta_{C}^{dr}$ OPs, the polymorphs were distinguished, and the essence of transformation was revealed.

According to classical nucleation theory (CNT) [85], the polymorphic transformation from *β*-HMX to *δ*-HMX is a process that begins with the nucleation of *δ*-HMX surrounding the *β*-HMX solid phase. Firstly, the number of *δ*-HMX molecules of dark blue increases, while the number of the *β*-HMX phase decreases, as shown by the decrease in the molecules in yellow; see the snapshots of (IA), (IIA), and (IIIA) in Figure 5. Then, the volume of *δ*-HMX becomes large enough to “touch” the edges of the periodic images, shown by (IB), (IIB), and (IIIB). In this process, the free energy gradually increases (see Figure 4), and in most cases, when the *δ*-HMX crystal nucleus touches the edges, it reaches the maximum in the PMF curve; see (IB), (IIB), and (IIIB). This indicates the size effect on the free energy [30,33]. Finally, the free energy decreases with the growth of *δ*-HMX.

According to the nucleation rate theory of CNT [43], when the rate of the *β*-HMX decomposition is equal to that of the *δ*-HMX growth, the system shows an equilibrium with a local minimum free-energy surface. As mentioned above, the formation of (IB), (IIB), and (IIIB) is caused by the size effect of the simulated box, and they are not the critical nucleus to form *δ*-HMX. From Figure 4, except for (IB), (IIB), and (IIIB), there is another transition state along the PMF path of the polymorphic transformation from *β*-HMX to *δ*-HMX shown in each of the curves, i.e., (ID), (IIC), or (IIID). Then, which should be the critical nucleus in the PMF? Here, an opposite process, i.e., *δ*-HMX → *β*-HMX, was examined. Different from *β*-HMX → *δ*-HMX, the real localized critical nucleation of *β*-HMX is found for *δ*-HMX → *β*-HMX, shown by a molecular cluster (in yellow) located in the middle of the simulation box. In other words, it was immersed in the dark blue *δ*-HMX space and not affected by the size effects, indicating that (ID) and (IIID) are representative of the *β*-HMX nucleus inside the *δ*-HMX crystal phase. This phenomenon has also been found in the other literature [68,86,87]. However, as for the snapshots (IIC) and (IID), due to the size effect, the yellow areas exceed the simulated box, and they cannot be determined as the critical nucleus.

Of course, if the simulation system is large enough, the *δ*-HMX critical nucleus can also be found with the higher energy barrier, indicating that the *β*-HMX → *δ*-HMX transformation is difficult, as is consistent with the experimental facts that *β*-HMX can be transformed into *δ*-HMX only at high temperatures [21,24].

### 2.4. Free Energy from Markovian Milestoning with Voronoi Tessellations

**Figure 6:**
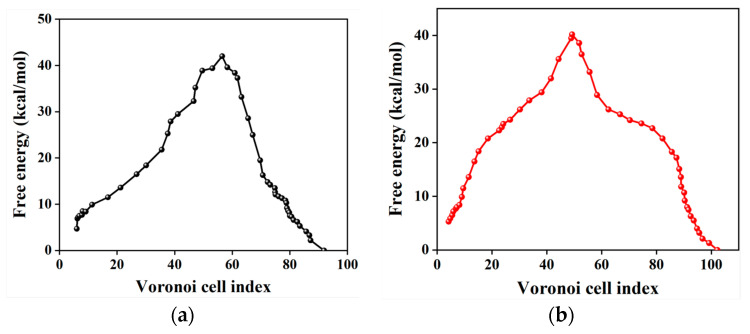
Free energy for the nucleation of polymorphic transformation from the Markovian milestoning with Voronoi tessellations. The left and right sides of the curve correspond to *δ*-HMX and *β*-HMX crystals. (**a**,**b**) are obtained from the string by K-means clustering sampling with $\theta_{C}^{db}$ and $\theta_{C}^{dr}$, respectively.

Figure 6 shows the free-energy profile along the MFEP of the polymorphic transformation between *δ*-HMX and *β*-HMX by the Voronoi milestoning procedure with order parameter $\theta_{C}^{dr}$ and $\theta_{C}^{dr}$. The activation free energies from $\theta_{C}^{dr}$ and $\theta_{C}^{dr}$ are close, 37.3 and 34.9 kcal/mol from *δ*-HMX to *β*-HMX, respectively. This once again indicates that the potential barrier of the transformation is not fundamentally dependent on the type of order parameter selected. From Figure 4 and Figure 6, the free energies obtained from the procedure of Markovian milestoning are lower than those from the PMF computed from the SMCV. Note that more entropy is removed in the SMCV simulations by restraining more degrees of freedom compared to the Markovian milestoning [77]. Furthermore, the MFEP from SMCV corresponds to a single pathway, while that from the milestoning calculation integrates over many pathways with a more accurate representation of the free energy involved in the polymorphic transformation [88,89,90,91,92,93,94,95,96,97,98].

**Figure 7:**
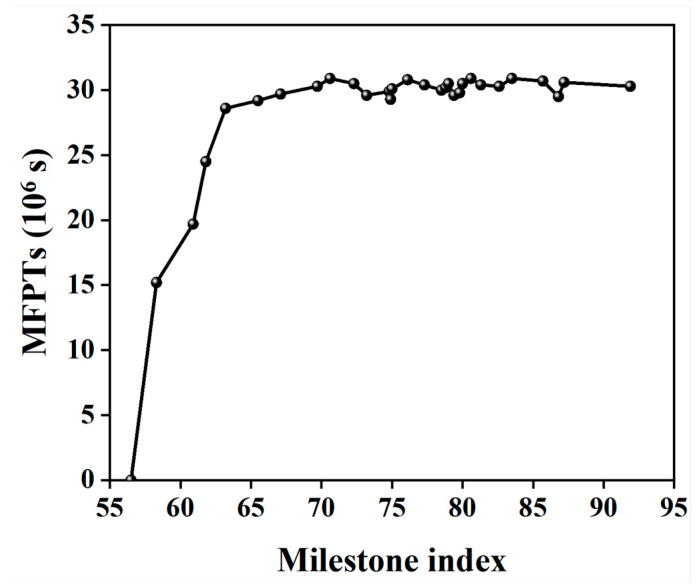
Mean first passage time to *β*-HMX.

According to Figure 7, the time of the nucleation for transformation from *δ*-HMX to *β*-HMX is calculated to be about 3.26 × 10^7^ s, and thus the obtained nucleation rate is about 4.0 × 10^17^ m^−3^·s^−1^ for the transformation.

### 2.5. Temperature Effect on Polymorphic Transformation

#### 2.5.1. Peaks in Pair Distribution Function

In order to clarify the temperature effect, except for 480 K of the temperature, the polymorphic transformation of HMX was also investigated at 510 K, 450 K, and 420 K, respectively. The average peak locations and concentration parameters are shown in Table S5. Compared with the values at 480 K in Table S3, the parameters of *β*-HMX and *δ*-HMX do not change obviously for *r* and 1/*σ*^2^, while for $\phi_{\hat{r}}$, ${\eta_{\hat{r}}}^{\alpha }$, $\phi_{q}$**,** and $\eta_{q}^{\alpha }$, significant changes are found. This result indicates that it is very necessary to introduce $\varphi_{\alpha ,i}^{\mathrm{db}}$ and $\varphi_{\alpha ,i}^{\mathrm{dr}}$ with the $\varphi_{\alpha ,i}^{b}$ and $\varphi_{\alpha ,i}^{r}$ components for the investigation of the temperature effect on the transformation, since, similar to the case at 480 K, the process of the transformation is obviously closely dependent on the $\varphi_{\alpha ,i}^{b}$ and $\varphi_{\alpha ,i}^{r}$ order parameters at different temperatures. For example, the transition between the chair-(*β*-HMX) and boat-shaped (*δ*-HMX) molecules of HMX is closely related to the temperature.

#### 2.5.2. Polymorphic Transformation from β-HMX to δ-HMX

The difference in the PMF between *β*-HMX and the transition state is 48.2, 55.8, and 63.5 kcal∙mol^−1^ with the $\theta_{C}^{db}$ as the collective variables at 510 K, 450 K, and 420 K, respectively. As for the $\theta_{C}^{dr}$, they are 51.3, 60.2, and 66.7 kcal∙mol^−1^, respectively. The difference in the PMF is decreased with the increase in the temperature. As mentioned above, in comparison with the free energy from Markovian milestoning, the values of the PMF are overestimated compared to the true value of the polymorphic transformation due to the reaction coordinates that are inconsistent with the real transformation path. Therefore, the temperature effect on the polymorphic transformation will not be discussed in detail by the PMF, but by the free energy from Markovian milestoning (see below).

**Figure 8:**
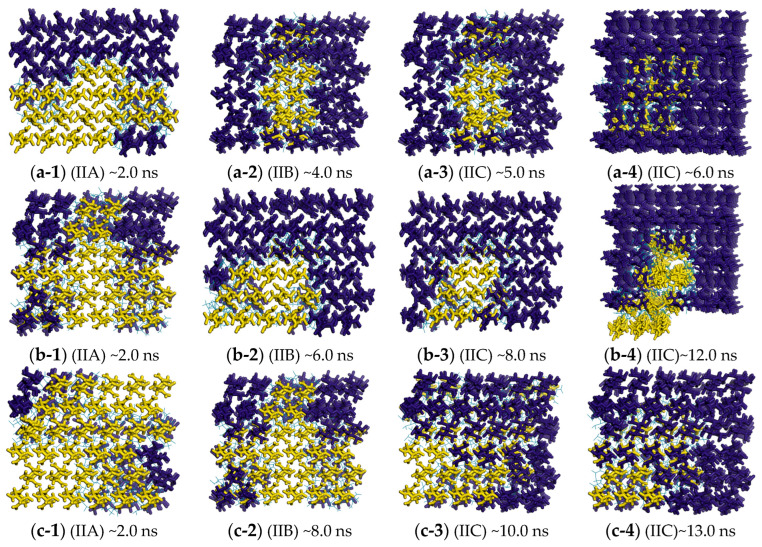
Changes in the local order parameters on the FTS path and the times for crystallization involving $\theta_{C}^{dr}$ with K-means clustering at different temperatures (“~” represents “approximately”). (IIA) ~2.0 ns (IIB) ~4.0 ns (IIC) ~5.0 ns ~6.0 ns (**a-1**–**a-4**) at 510 K; (IIA) ~2.0 ns (IIB) ~6.0 ns (IIC) ~8.0 ns ~12.0 ns (**b-1**–**b-4**) at 450 K; (IIA) ~2.0 ns (IIB) ~8.0 ns (IIC) ~10.0 ns ~13.0 ns (**c-1**–**c-4**) at 420 K.

The local $\theta_{C}^{dr}$ OPs were shown for the key snapshots of the polymorphic transformation at the different temperatures (see Figure 8). At all the temperatures, the molecule changes from the “all yellow” to “complete dark blue” conformation along the trajectory of *β*-HMX → (IIA) → (IIB) → (IIC) → *δ*-HMX, companied by a complete transformation. This shows that, similar to the cases at 480 K, polymorphic transformation is also a locally initiated process at any temperature.

From Figure 5 (II) and Figure 8, the time of the polymorphic transformation from *β*-HMX to *δ*-HMX is increased with the decrease in the temperature. This is shown by that, after the same time, the composition of the dark blue molecules decreases, accompanied by a decrease in the conversion into *δ*-HMX as the temperature decreases. For key points on the PFM curve, such as transition states or intermediates, the time prolongs as the temperature decreases, indicating an increase in the time required for conversion to the same stage. The times of the polymorphic transformation are 6 ns, 8 ns, 12 ns, and 13 ns at 510 K, 480 K, 450 K, and 420 K, respectively. The times of the polymorphic transformation at the stage of (C) (i.e., the highest point on the curve of PMF) are 4 ns, 4 ns, 6 ns, and 8 ns at 510 K, 480 K, 450 K, and 420 K, respectively. As the temperature decreases, the motion of atoms and molecules weakens, accompanied by the slow changes in the bond orientation and relative orientation OPs, resulting in the long time of the polymorphic transformation. Note that the polymorphic transformation often occurs at a suitable temperature, rather than at higher or lower temperatures. In fact, the temperatures selected in this study are close to the real temperature range in the experiment (438.0~583.0 K [20]).

**Figure 9:**
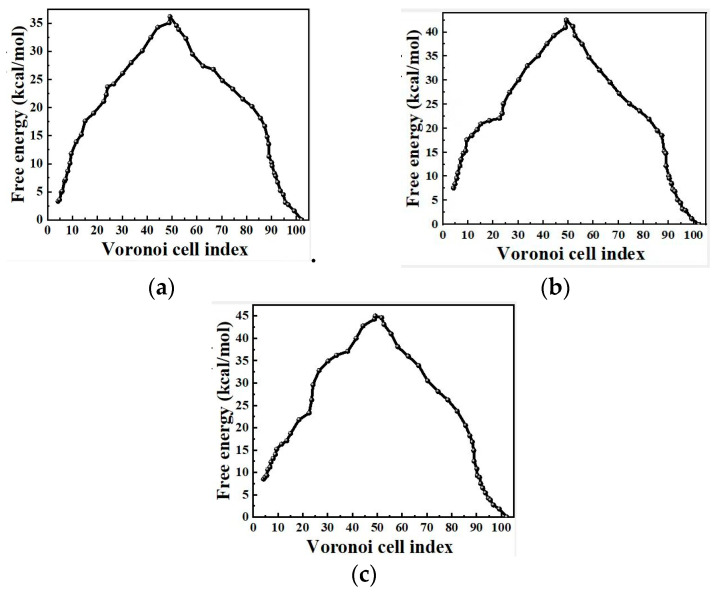
Free energy obtained by Markovian milestoning with Voronoi tessellations at different temperatures, with K-means clustering sampling. (**a**) 510 K, (**b**) 450 K, (**c**) 420 K.

The free energy associated with the nucleation of the polymorphic transformation is obtained from the simulations using Markovian milestoning with Voronoi tessellations by order parameter $\theta_{C}^{dr}$ at 510 K, 450 K, and 420 K, respectively (see Figure 9). The higher the temperature, the lower the free-energy barrier becomes. They are 36.2, 40.2, 42.5 and 45.0 kcal/mol at 510 K, 480 K, 450 K, and 420 K, respectively. According to our calculations, the higher the temperature, the smaller the free energies of *β*-HMX and transition states are, which is consistent with the fundamental thermodynamic principle of Δ*G* = Δ*H* − *T*Δ*S*, where *T*, Δ*G*, Δ*H*, and Δ*S* are the temperature, free-energy change, enthalpy change, and entropy change, respectively. Obviously, in the process of nucleation in polymorphic transformation, Δ*S* corresponding to the transition state is always greater than zero. Therefore, as the temperature increases, the Δ*G* value of the transition state will decrease, and the corresponding free energies of *β*-HMX will also decrease since the free-energy barrier decreases. In other words, as the temperature increases, both the free energies of *β*-HMX and the transition state decrease. Due to the more significant decrease in the free energy of the transition state, the value of the free-energy barrier decreases (note: the relative values shown in Figure 9). This indicates that, as the temperature increases, it is beneficial for the polymorphic transformation from *β*-HMX to *δ*-HMX, as is in accordance with the previous experimental results [20].

To further reveal the essence of the transformation orientation controlled by the temperature, the pre-exponential factor and nucleation rate are discussed. According to $r*\approx (Nk_{B}T/h)exp(-\Delta G*/k_{B}T)$ [99], where $\left(Nk_{B}T/h\right)$ and ΔG* are the pre-exponential factor and the free-energy barrier of nucleation, the rate of nucleation of the polymorphic transformation is increased as the temperature increases. Except for the decreased free-energy barrier mentioned above, the value of the pre-exponential factor is increased as the temperature increases, which also facilitates the transformation towards *δ*-HMX. When the temperature increases, the number of molecules colliding with each other at the nucleation interface increases, leading to an increased probability of nucleation, and thus an increase in the pre-exponential factor.

**Figure 10:**
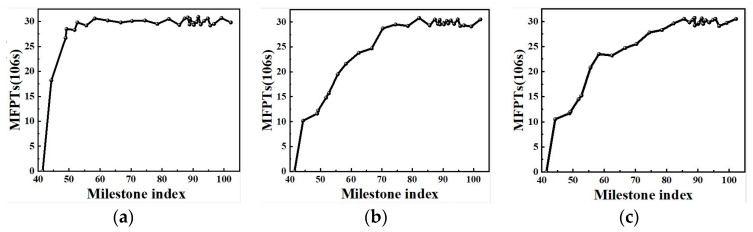
Mean first passage time to *β*-HMX at different temperatures. (**a**) 510 K, (**b**) 450 K, (**c**) 420 K.

From Figure 10, as the temperature increases, the milestone index corresponding to the mean first passage time decreases obviously.

### 2.6. Prediction of Impact Sensitivity for HMX Polymorph

In the past 20 years, the impact sensitivity has been extensively predicted by the bond dissociation energies [100], electron density topologies [101], crystal void space and compressibility [102], and electronic band gaps [103]. All of these methods tend to offer a more qualitative rationale for impact sensitivity. In contrast, vibrational up-pumping has emerged as a reliable tool capable of successfully ranking a broad range of energetic materials according to their experimental impact sensitivity values [103,104,105,106,107,108], i.e., the impact sensitivity is well correlated with the total up-pumping into internal vibrational modes. In particular, they found that impact sensitivity predictions based on the vibrational up-pumping model show a strong polymorph dependency, and the origin of the predicted impact sensitivity variation can be attributed to the vibrational mode and to differences in the molecular conformation for polymorphs [105,106,107].

From Figure 1a it can be seen that the six-membered ring of *β*-HMX is a chair-shaped structure, while that of *δ*-HMX is a boat-shaped structure. In the molecular structure of *β*-HMX, four nitro groups are approximately symmetrical in the dislocation form, and the distribution of them is relatively dispersed, while in the molecular structure of *δ*-HMX, all the nitro groups face in one direction, presenting a crowded state. According to the definition of the vibrational up-pumping [103,104,105,106,107,108], it is evident that the up-pumping value of *δ*-HMX should be greater than that of *β*-HMX. According to the literature [105] of Christopher and Morrison et al., under the external pressure, the molecular conformation of *γ*-RDX is more crowded with the nitro groups compared to other RDX molecules, resulting in a stronger internal vibration, interaction, and larger up-pumping value. The up-pumping process localizes the initial mechanical energy through the external vibrations into the local modes, resulting in the activation of trigger linkages, and bond breaking and initiation, and thus the more sensitive a material is to impact (i.e., the lower the mechanical stimulus needed to initiate the energetic materials), the higher the calculated up-pumped density [105]. Therefore, the impact sensitivity of *β*-HMX is smaller than that of *δ*-HMX, making *δ*-HMX more prone to explosion under impact, which is consistent with the previous experimental results [21,22,25].

## 3. Theory

### 3.1. Order Parameters

Via the structural variables, such as the centers of mass *r_ij_*, bond orientation $\phi_{\hat{r}}$, and relative orientation $\phi_{q}$ between molecules, the simple OPs (i.e., $\varphi_{\alpha ,i}^{d}$, $\varphi_{\alpha ,i}^{b}$, and $\varphi_{\alpha ,i}^{r}$) and combined OPs (i.e., $\varphi_{\alpha ,i}^{\mathrm{db}}$ and $\varphi_{\alpha ,i}^{\mathrm{dr}}$) can be built as follows:

(1) $$\varphi_{\alpha ,i}^{d}=\sum_{j\neq i}\frac{1}{\sqrt{2\pi }\sigma_{\alpha }}\exp\left[-\frac{{\left({\hat{r}}_{ij}-{\hat{r}}_{\alpha }\right)}^{2}}{2\sigma_{\alpha }^{2}}\right]\;$$

(2) $$\varphi_{\alpha ,i}^{b}=\sum \frac{1}{2\pi I_{0}\left({\eta_{\hat{r}}}^{\alpha }\right)}\exp\left[{\eta_{\hat{r}}}^{\alpha }\cos2\left(\phi_{\hat{r}}-\phi_{\hat{r}}^{\alpha }\right)\right]\;$$

(3) $$\varphi_{\alpha ,i}^{r}=\sum \frac{1}{2\pi I_{0}\left(\eta_{q}^{\alpha }\right)}\exp\left[\eta_{q}^{\alpha }\cos2\left(\phi_{q}-\phi_{q}^{\alpha }\right)\right]\;$$

(4) $$\varphi_{\alpha ,i}^{\mathrm{db}}=\frac{1}{\sqrt{2\pi }\sigma_{\alpha }}\frac{1}{2\pi I_{0}\left({\eta_{\hat{r}}}^{\alpha }\right)}\sum_{j\neq i}\exp\left[-\frac{{\left({\hat{r}}_{ij}-{\hat{r}}_{\alpha }\right)}^{2}}{2\sigma_{\alpha }^{2}}\right]\exp\left[{\eta_{\hat{r}}}^{\alpha }\cos2\left(\phi_{\hat{r}}-\phi_{\hat{r}}^{\alpha }\right)\right]\;$$

(5) $$\varphi_{\alpha ,i}^{\mathrm{dr}}=\frac{1}{\sqrt{2\pi }\sigma_{\alpha }}\frac{1}{2\pi I_{0}\left(\eta_{q}^{\alpha }\right)}\sum_{j\neq i}\exp\left[-\frac{{\left({\hat{r}}_{ij}-{\hat{r}}_{\alpha }\right)}^{2}}{2\sigma_{\alpha }^{2}}\right]\exp\left[\eta_{q}^{\alpha }\cos2\left(\phi_{q}-\phi_{q}^{\alpha }\right)\right]\;$$

where ${\hat{r}}_{\alpha }$, $\phi_{\hat{r}}^{\alpha }$, and $\phi_{q}^{\alpha }$ denote the mean center-of-mass distance vector, bond orientation, and relative orientation corresponding to the peak α, *σ_α_*, ${\eta_{\hat{r}}}^{\alpha }$, and $\eta_{q}^{\alpha }$ are the corresponding standard deviation and concentration parameters, and *I*_0_ is the modified Bessel function [41].

To simplify the collective variables, the local OPs are often adopted. One is the OP for a molecule *i* with all the *α* peaks throughout the system (i.e., $\theta_{i}^{*}$, “*” represents “db” or “dr”), and the other is the averaged OP for all the molecules within the divided cell (*C*) with all the *α* peaks (i.e., $\theta_{C}^{*}$).

(6) $$\theta_{i}^{*}=\sum_{\alpha }\varphi_{i,\alpha }^{*}$$

(7) $$\theta_{C}^{*}=\frac{1}{N_{C}}\sum_{i\in C}\sum_{\alpha }\varphi_{i,\alpha }^{*}\;$$

### 3.2. String Method

String method means a technique by which an MFEP can be determined by evaluating the mean force and its tensor with the collective variables [57].

For a string *z*(α, *t*), where *t* is the evolutionary time and α ∈ [0, 1], the equation by which the free-energy gradient $\frac{\partial F\left(z\left(\alpha ,t\right)\right)}{\partial z_{k}}$ can be evolved and converged to an MFEP is as follows:

(8) $$\frac{\partial z_{i}\left(\alpha ,t\right)}{\partial t}=-\sum_{j,k=1}^{N}P_{ij}\left(\alpha ,t\right)M_{jk}\left(z\left(\alpha ,t\right)\right)\frac{\partial F\left(z\left(\alpha ,t\right)\right)}{\partial z_{k}}\;$$

where *P_ij_*(α, *t*) is the projector on the plane perpendicular to the path at *z*(α, *t*), *M_jk_*(*z*(α, *t*)) is the tensor.

Given two minima, i.e., *z*(0, *t*) = *z_a_* and *z*(1, *t*) = *z_b_*, when *t →* ∞, the solution of the above equation converges to an MFEP, and the corresponding tangent vector will be parallel to M(z)∇F(z), i.e.,

(9) $$0=\sum_{j,k=1}^{N}P_{ij}\left(\alpha \right)M_{jk}\left(z\left(\alpha \right)\right)\frac{\partial F\left(z\left(\alpha \right)\right)}{\partial z_{k}}\;$$

where $P_{ij}\left(\alpha \right)=\delta_{ij}-{\hat{t}}_{i}\left(\alpha \right){\hat{t}}_{j}\left(\alpha \right)$, ${\hat{t}}_{i}\left(\alpha \right)=\frac{\partial z_{i}/\partial \alpha }{\left|\partial z_{i}/\left.\partial \alpha \right|\right.}\;$, and *M_jk_*(*z*) is given by

(10) $$M_{ij}\left(z\right)=Z^{-1}e^{\beta F\left(z\right)}\int_{R^{n}}\sum_{k=1}^{n}\frac{\partial \theta_{i}\left(x\right)}{\partial x_{k}}\frac{\partial \theta_{j}\left(x\right)}{\partial x_{k}}e^{-\beta V\left(x\right)}\delta \left(z_{1}-\theta_{1}\left(x\right)\right)\ldots \delta \left(z_{N}-\theta_{N}\left(x\right)\right)dx\;$$

when *k →* ∞ with the large enough *T*, according to Equations (9) and (10),

(11) $$\frac{\partial F\left(z\right)}{\partial z_{j}}\approx \frac{k}{T}\int_{0}^{T}\left(z_{j}-\theta_{j}\left(x\left(t\right)\right)\right)dt$$

(12) $$M_{\mathrm{ij}}\left(z\right)\approx \frac{1}{T}\sum_{k=1}^{n}\int_{0}^{T}\frac{\partial \theta_{i}\left(x\left(t\right)\right)}{\partial x_{k}}\frac{\partial \theta_{j}\left(x\left(t\right)\right)}{\partial x_{k}}dt$$

### 3.3. Determining MFEP by FTS

#### 3.3.1. Initial Trajectory

The first step in determining an MFEP is to build an initial string trajectory connecting *z_a_* and *z_b_*. The initial OPs $\theta_{i,m}^{*}\left(0\right)$ and $\theta_{C,m}^{*}\left(0\right)$ can be calculated from an MD simulation of each initial replica in the string, where “m” means the m*th* replica.

#### 3.3.2. K-Means Clustering

The second step is to obtain an initial smooth string via the K-means clustering algorithm. The restricting sampling was adopted by the harmonic functions:

(13) $$\psi \left(\theta_{C}^{d,b}\right)=\frac{k_{d}}{2}{\left[\theta_{C,m}^{d}-\theta_{C,m}^{d}\left(0\right)\right]}^{2}+\frac{k_{b}}{2}{\left[\theta_{C,m}^{b}-\theta_{C,m}^{b}\left(0\right)\right]}^{2}$$

(14) $$\psi \left(\theta_{C}^{d,r}\right)=\frac{k_{d}}{2}{\left[\theta_{C,m}^{d}-\theta_{C,m}^{d}\left(0\right)\right]}^{2}+\frac{k_{r}}{2}{\left[\theta_{C,m}^{r}-\theta_{C,m}^{r}\left(0\right)\right]}^{2}$$

where *k^d^*, *k^b,^* and *k^r^* mean the spring coefficients.

#### 3.3.3. Determining MFEP by SMCV

The steps to determine the MFEP by SMCV are as follows:

*Step 1* According to Equations (11) and (12), the values of $\nabla_{z}F(z_{C,m}^{*})$ and $M(z_{C,m}^{*})$ are estimated.

*Step 2* Via the string evolution equation [84], a target OPs $z_{C,m(new)}^{*}$ is calculated.

(15) $$z_{C,m(new)}^{*}=z_{C,m}^{*}-\Delta \tau M\left(z_{C,m}^{*}\right)\nabla F\left(z_{C,m}^{*}\right)$$

*Step 3* Interpolate a curve *z*(α) through $z_{C,m(new)}^{*}$ by the b-spline fitting method, and a new target OP $z_{C,m+1}^{*}$ is calculated.

*Step 4* Obtaining MFEP.

*Step 5* The OPs and PMF are calculated.

### 3.4. Markovian Milestoning with Voronoi Tessellations

An accurate free-energy profile could be obtained from Markovian milestoning with Voronoi tessellations [55,77,78,79].

#### 3.4.1. Construction of Markovian Milestoning with Voronoi Tessellations

The tessellations are defined in the OP space for each replica, and the MFEP form is projected by using principal component analysis (PCA) [109]. Then, an MD simulation is performed for each replica within their cells, and the planar half-pseudoharmonic restraining potentials [35] are used to keep the images within their cells.

#### 3.4.2. Accumulating Statistics of the Number N_i,j_, $N_{a,b}^{i}$, and $T_{a}^{i}$

Accumulate statistics of the number *N*_i,j_ of collisions by testing the condition of $\left\|\theta (r)-z_{j}\right\|<\left\|\theta (r)-z_{i}\right\|$ at time *t* + δ*t* and $\left\|\theta (r)-z_{j}\right\|>\left\|\theta (r)-z_{i}\right\|$ at time *t*, $N_{a,b}^{i}$ and the total time $T_{a}^{i}$ were counted.

#### 3.4.3. Calculating the Probabilities π_i_ and Free Energy F_i_

According to the probabilities *π*_i,_ the free energy *F*_i_ is calculated by *F*_i_ = −k*T*ln*π*_i_. *π*_i_ can be obtained at steady state given by

(16) $$\frac{d\pi_{i}}{dt}=\sum_{j\neq i}(\pi_{j}v_{j,i}-\pi_{i}v_{i,j})=0,\;i,j=1,\ldots ,N,$$

where *ν*_i,j_ is the rate of escape.

#### 3.4.4. Calculating Mean First Passage Times (MFPTs)

MFPTs *T*_a,b∗_ to a reference milestone *b*^∗^ can be estimated as follows

(17) $$\sum_{b\neq b*}k_{a,b}T_{b,b*}=-1$$

where $k_{a,b}$ is the rate of instantaneous transition from milestone *a* to milestone *b*. Then, the nucleation rate was calculated.

## 4. MD Simulation Details

According to the symmetry of the ring frame, the direction of the axis connecting the center of two six-membered rings of HMX was used as an approximate measure of the *r_ij_* between the centers of mass of two HMX molecules; see Figure 2.

Firstly, a short MD simulation was carried out for the structure of *β*-HMX or *δ*-HMX from the experimental crystal at 1 bar and 480 K, which is close to the experimental temperature of transformation (438~483 K [20]). Then, a 2.5 ns MD simulation was carried out at 480 K and constant volume. The CHARMM22 force field [98,99] was used in the NAMD software package [110,111]. The simulations were performed using a time step of 0.5 fs, a Langevin thermostat with a damping constant of 25 ps^−1^, and a Langevin piston barostat with a damping time scale of 50 fs. Electrostatics were handled using the particle mesh Ewald method [112] with a cutoff of 12 Å.

According to the literature [113], a non-physical method has been applied and a trajectory initiated from the *β*-HMX basin with 216 molecules was found to transform into *δ*-HMX over several ns, judged by the ratio of the **b** to **c** lattice vectors (**b**:**c** ≈ 5:4 for *β*-HMX and **b**:**c** ≈ 1:4.5 for *δ*-HMX). Thus, an initial string with 22 replicas (including *β*-HMX and *δ*-HMX basins) was confirmed based on the approximately equal **b**/**c** step size.

In the SMCV simulations with FTS as well as the calculations of free energy from Markovian milestoning with Voronoi tessellations, the setting was the same as that in the construction of OPs. All the calculations were implemented by using PLUMED package [114].

The temperature effects on the polymorphic transformation of HMX were simulated at 510 K, 450 K, and 420 K, respectively. The simulation details are consistent with those at 480 K.

## 5. Conclusions

In this work, a K-means clustering algorithm for the constructed OPs of HMX was used to optimize the string. The MFEP and PMF for the transformation between *β*-HMX and *δ*-HMX were obtained by the SMCV method. The free-energy profile was constructed and the influences of the temperature on the nucleation were also investigated.

(1) The K-means clustering algorithm is suitable to enhance the sampling of the OPs in revealing the polymorphic transformation for the molecular crystals of HMX with a ring-like molecular structure by FTS. The convergence of the $\theta_{C}^{d}$, $\theta_{C}^{b}$, or $\theta_{C}^{r}$ order parameter as the collective variable is difficult, while for the combined $\theta_{C}^{db}$ and $\theta_{C}^{dr}$ OPs with the K-means clustering, the strings were converged quickly. This confirms the effectiveness and advantage of the K-means clustering $\theta_{C}^{db}$ and $\theta_{C}^{dr}$ as the collective variables for FTS.

(2) The polymorphic transformation could be regarded as a process of the nucleation of *β*-HMX within the *δ*-HMX phase. The barriers of transformation were independent on OP types but affected by the sampling algorithm models and finite-size effects. The configuration with the lower potential barrier in PMF corresponded to the critical nucleus.

(3) As the temperature increases, the time and free-energy barrier of the polymorphic transformation were reduced, which was explained by the pre-exponential factor and nucleation rate. This polymorphic transformation of HMX was controlled by the temperatures. As the temperature increases, the milestone index corresponding to the mean first passage time decreases obviously.

(4) According to the inspiration of the vibrational up-pumping by Christopher and Morrison, the impact sensitivity of *β*-HMX should be smaller than that of *δ*-HMX, making *δ*-HMX more prone to explosion under impact, which is consistent with the previous experimental results.

This work is useful in screening high-energetic and insensitive explosives in industrial production.

## Data Availability

The data related to this research can be accessed upon reasonable request via email.

## Supplementary Materials

The following supporting information can be downloaded at: https://www.mdpi.com/article/10.3390/molecules29204819/s1, Table S1: lattice parameters of *β*-HMX and *δ*-HMX, Table S2: peaks in the pair distribution function for the reference structures of the *β*-HMX and *δ*-HMX crystals, Table S3: average peak locations and concentrations parameters for the *β*-HMX and *δ*-HMX crystals, Table S4: average peak locations for the replicas by average-based sampling at 480 K, Table S5: the values of the partition coefficient and partition entropy, Table S6: average peak locations and concentrations parameters for the *β*-HMX and *δ*-HMX crystals at 510 K, 450 K, and 420 K.

## References

[1] Tai B.T., Andrew J.S., David A.K. (2011). Efficient calculation of α- and β-nitrogen free energies and coexistence conditions via overlap sampling with targeted perturbation. J. Chem. Phys..

[2] Cardew P.T., Davey R.J., Ruddick A.J. (1984). Kinetics of polymorphic solid-state transformations. J. Chem. Soc. Faraday Trans..

[3] Davey R.J., Maginn S.J., Andrews S.J., Buckley A.M., Cottier D., Dempsay P., Rout J.E., Stanley D.R., Taylor A. (1993). Stabilization of a metastable crystalline phase by twinning. Nature.

[4] Morris K.R., Griesser U.J., Eckhardt C.J., Stowell J.G. (2001). Theoretical approaches to physical transformations of active pharmaceutical ingredients during manufacturing processes. Adv. Drug Delivery Rev..

[5] Zhang G.G.Z., Gu C., Zell M.T., Burkhardt R.T., Munson E.J., Grant D.J.W. (2002). Crystallization and Transitions of Sulfamerazine Polymorphs. J. Pharm. Sci..

[6] Kim Y.S., Paskow H.C., Rousseau R.W. (2005). Propagation of solid-state transformations by dehydration and stabilization of pseudopolymorphic crystals of sodium naproxen. Cryst. Growth Des..

[7] Herbstein F.H. (2006). On the mechanism of some first-order enantiotropic solid-state phase transitions: From Simon through Ubbelohde to Mnyukh. Acta Crystallogr..

[8] Sadovnikov S.I., Gusev A.I., Chukin A.V., Rempel A.A. (2016). High-temperature X-ray diffraction and thermal expansion of nanocrystalline and coarse-crystalline acanthite α-Ag_2_S and argentite β-Ag_2_S. Phys. Chem. Chem. Phys..

[9] Gonzalez S.D., Vega C. (2010). Melting point and phase diagram of methanol as obtained from computer simulations of the OPLS model. J. Chem. Phys..

[10] Wei X.F., Xu J.J., Li H.Z., Long X.P., Zhang C.Y. (2016). Comparative study of experiments and calculations on the polymorphisms of 2,4,6,8,10,12-hexanitro-2,4,6,8,10,12-hexaazaisowurtzitane (CL-20) precipitated by solvent/antisolvent method. J. Phys. Chem. C.

[11] Irie M., Kobatake S., Horichi M. (2001). Reversible surface morphology changes of a photochromic diarylethene single crystal by photoirradiation. Science.

[12] Vippagunta S.R., Brittain H.G., Grant D.J.W. (2001). Crystalline solids. Adv. Drug Delivery Rev..

[13] Tonauer C.M., Bauer M., Loerting T. (2022). The impact of temperature and unwanted impurities on slow compression of ice. Phys. Chem. Chem. Phys..

[14] Yi P., Falk M.L., Weihs T.P. (2017). Suppression of homogeneous crystal nucleation of the NiAl intermetallic by a composition gradient: A molecular dynamics study. J. Chem. Phys..

[15] Bernshtein J. (2002). Polymorphism in Molecular Crystals.

[16] Sikder A.K., Sikder N. (2004). A review of advanced high performance, insensitive and thermally stable energetic materials emerging for military and space applications. J. Hazard. Mater..

[17] Sivabalan R., Gore G.M., Nair U.R., Saikia A., Venugopalan S., Gandhe B.R. (2007). Study on ultrasound assisted precipitation of CL-20 and its effect on morphology and sensitivity. J. Hazard. Mater..

[18] Vrcelj R.M., Gallagher H.G., Sherwood J.N. (2001). Polymorphism in 2,4,6-Trinitrotoluene Crystallized from Solution. J. Am. Chem. Soc..

[19] Parrish D.A., Deschamps J.R., Gilardi R.D., Butcher R.J. (2008). Polymorphs of Picryl Bromide. Cryst. Growth Des..

[20] Cady H.H., Smith L.C. (1962). Studies on the Polymorphs of HMX.

[21] Smilowitz L.B., Henson B.F., Asay B.W. (2006). Interfacial and volumetric kinetics of the β→δ phase transition in the energetic nitramine octahydro-l,3,5,7-tetranitro-l,3,5,7-tetrazocine based on the virtual melting mechanism. J. Chem. Phys..

[22] Smilowitz L.B., Henson B.F., Asay B.W. (2001). Kinetics of the β→δ phase transition in PBX9501. Shock. Campression Condens. Matter..

[23] Brill T.B., Karpowicz R.J. (1982). Solid phase transition kinetics The role of intermolecular forces in the condensed-phase decamposition of octahydro-l,3,5,7-tetranitro-l,3,5,7-tetrazocine. J. Phys. Chem..

[24] Weese R.K. (2000). Kinetics of β→δ Solid-Solid Phase Transition of HMX. UCRL-LR-143960.

[25] Smilowitz L.B., Henson B.F., Asay B.W., Dickson P. (2002). The β→δ phase transition in the energetic nitramine octahydmo-l,3,5,7-tetranitmo-l,3,5,7-tetrazocine. Kinetics. J. Chem. Phys..

[26] Bao J.N., Fan H.B., Xue X.J., Xie Q., Pan P.J. (2018). Temperature-dependent crystalline structure and phase transition of poly(butylene adipate) end-functionalized by multiple hydrogen-bonding groups. Phys. Chem. Chem. Phys..

[27] Handle P.H., Loerting T. (2015). Temperature-induced amorphisation of hexagonal ice. Phys. Chem. Chem. Phys..

[28] Diana H., Pilar C., Rico G., Benjamin W., Rajadurai C., Svetlana K., Mario R., Roberto-Carlos S., Klaus K., Doris G. (2018). Polymorphism and metal-induced structural transformation in 5,5′-bis(4-pyridyl)(2,2′-bispyrimidine) adlayers on Au(111). Phys. Chem. Chem. Phys..

[29] Venugopal K., Deepak D., Suresh D., Sunil V. (2015). Transformation of photophysical properties from solution to solid state in alkoxy-cyano-diphenylacetylene molecules. Phys. Chem. Chem. Phys..

[30] Beckham G.T., Peters B., Trout B.L. (2008). Evidence for a size dependent nucleation mechanism in solid state polymorph transformations. J. Phys. Chem. B.

[31] Mnyukh Y.V. (1976). Polymorphic transitions in crystals: Nucleation. J. Cryst. Growth.

[32] Tuble S.C., Anwar J., Gale J.D. (2004). An Approach to developing a force field for molecular simulation of martensitic phase transitions between phases with subtle differences in energy and structure. J. Am. Chem. Soc..

[33] Beckham G.T., Peters B., Starbuck C., Variankaval N., Trout B.L. (2007). Surface-mediated nucleation in the solid-state polymorph transformation of terephthalic acid. J. Am. Chem. Soc..

[34] Bayés-García L., Calvet T., Cuevas-Diarte M.À., Ueno S., Sato S. (2013). Crystallization and transformation of polymorphic forms of trioleoyl glycerol and 1,2-dioleoyl-3-rac-linoleoyl glycerol. J. Phys. Chem. B.

[35] Santiso E.E., Trout B.L. (2015). A general method for molecular modeling of nucleation from the melt. J. Chem. Phys..

[36] Maddox J. (1995). Colloidal crystals model real world. Nature.

[37] Price S.L. (2009). Computed crystal energy landscapes for understanding and predicting organic crystal structures and polymorphism. Acc. Chem. Res..

[38] Price S.L. (2008). From crystal structure prediction to polymorph prediction: Interpreting the crystal energy landscape. Phys. Chem. Chem. Phys..

[39] Dickson A., Warmflash A., Dinner A.R. (2009). Nonequilibrium umbrella sampling in spaces of many order parameters. J. Chem. Phys..

[40] Faradjian A.K., Elber R. (2004). Computing time scales from reaction coordinates by milestoning. J. Chem. Phys..

[41] Santiso E.E., Trout B.L. (2011). A general set of order parameters for molecular crystals. J. Chem. Phys..

[42] Carter E., Ciccotti G., Hynes J., Kapral R. (1989). Constrained reaction coordinate dynamics for the simulation of rare events. Chem. Phys. Lett..

[43] Anwar J., Zahn D. (2011). Uncovering molecular processes in crystal nucleation and growth by using molecular simulation. Angew. Chem. Int. Ed..

[44] Dan S., Mondal B., Saha S.K., Mondal S., Ranganathan R., Kumar M., Mazumdar C. (2022). Similar and dissimilar properties of polymorphic phases of NdIr_3_. J. Phys. Chem. C.

[45] Bellucci M.A., Trout B.L. (2014). Bézier curve string method for the study of rare events in complex chemical systems. J. Chem. Phys..

[46] Ovchinnikov V., Karplus M. (2014). Investigations of α-helix↔β-sheet transition pathways in a miniprotein using the finite-temperature string method. J. Chem. Phys..

[47] Olsson T.S., Ladbury J.E., Pitt W.R., Williams M.A. (2011). Extent of enthalpy–entropy compensation in protein–ligand interactions. Protein Sci..

[48] Berendsen H.J. (1998). A Glimpse of the Holy Grail?. Science.

[49] Branduardi D., Gervasio F.L., Parrinello M. (2007). From A to B in free energy space. J. Chem. Phys..

[50] Laio A., Parrinello M. (2002). Escaping free-energy minima. Proc. Natl. Acad. Sci. USA.

[51] Park S., Khalili-Araghi F., Tajkhorshid E., Schulten K. (2003). Free energy calculation from steered molecular dynamics simulations using Jarzynski’s equality. J. Chem. Phys..

[52] Weinan E., Ren W., Vanden-Eijnden E. (2002). String method for the study of rare events. Phys. Rev. B Condens. Matter Mater. Phys..

[53] Kulshrestha A., Punnathanam S.N., Ayappa K.G. (2022). Finite temperature string method with umbrella sampling using path collective variables: Application to secondary structure change in a protein. Soft Matter..

[54] Weinan E., Ren W., Vanden-Eijnden E. (2005). Finite temperature string method for the study of rare events. J. Phys. Chem. B.

[55] Vanden-Eijnden E., Venturoli M. (2009). Revisiting the finite temperature string method for the calculation of reaction tubes and free energies. J. Chem. Phys..

[56] Jónsson H., Mills G., Jacobsen K.W. (1998). Nudged elastic band method for finding minimum energy paths of transitions. Class. Quantum Dyn. Condens. Phase Simul..

[57] Maragliano L., Fischer A., Vanden-Eijnden E., Ciccotti G. (2006). Learning Markovian dynamics with spectral maps. J. Chem. Phys..

[58] Ren W., Vanden-Eijnden E., Maragakis P., Weinan E. (2005). Nonlinear discovery of slow molecular modes using state-free reversible VAMPnets. J. Chem. Phys..

[59] Zinovjev K., Tuñón I. (2017). Adaptive finite temperature string method in collective variables. J. Phys. Chem. A.

[60] Dickson B.M., Huang H., Post C.B. (2012). Unrestrained computation of free energy along a path. J. Phys. Chem. B.

[61] Díaz L.G., Ensing B. (2012). Path finding on high-dimensional free energy landscapes. Phys. Rev. Lett..

[62] Maragliano L., Roux B., Vanden-Eijnden E. (2014). Comparison between mean forces and swarms-of-trajectories string methods. J. Chem. Theory Comput..

[63] Song H.D., Zhu F.Q. (2017). Morphology evolution of polymer blends under intense shear during high speed thin-wall injection molding. J. Phys. Chem. B.

[64] Cao L.R., Lv C., Yang W. (2013). Hidden conformation events in DNA base extrusions: A generalized-ensemble path optimization and equilibrium simulation study. J. Chem. Theory Comput..

[65] Badaoui M., Buigues P.J., Berta D., Mandana G.M., Gu H., Földes T., Dickson C.J., Hornak V., Kato M., Molteni C. (2022). Combined free-energy calculation and machine learning methods for understanding ligand unbinding kinetics. J. Chem. Theory Comput..

[66] He X.X., Shen Y., Hung F.R., Santiso E.E. (2015). Molecular simulation of homogeneous nucleation of crystals of an ionic liquid from the melt. J. Chem. Phys..

[67] Salvalaglio M., Vetter T., Giberti F., Mazzotti M., Parrinello M. (2012). Uncovering molecular details of urea crystal growth in the presence of additives. J. Am. Chem. Soc..

[68] Shah M., Santiso E.E., Trout B.L. (2011). Computer simulations of homogeneous nucleation of benzene from the melt. J. Phys. Chem. B.

[69] Gobbo G., Bellucci M.A., Tribello G.A., Ciccotti G., Trout B.L. (2018). Nucleation of molecular crystals driven by relative information entropy. J. Chem. Theory Comput..

[70] Ren F.D., Liu Y.Z., Ding K.W., Chang L.L., Cao D.L., Liu S.B. (2024). Finite temperature string by K-means clustering sampling with order parameter as collective variables for molecular crystal: Application to polymorphic transformation between β-CL-20 and ε-CL-20. Phys. Chem. Chem. Phys..

[71] Ren F.D., Wang X.L., Zhang Q., Wang X.J., Chang L.L., Zhang Z.T. (2024). Experimental and theoretical investigation of external electric-field-induced crystallization of TKX-50 from solution by finite-temperature string with order parameters as collective variables for ionic crystals. Molecules.

[72] Adelman J.L., Grabe M. (2013). Simulating rare events using a weighted ensemble-based string method. J. Chem. Phys..

[73] Mac Q.J. (1967). Some Methods for Classification and Analysis of Multivariate Observations.

[74] Nawaz M., Mehmood Z., Nazir T., Naqvi R.A., Rehman A., Iqbal M., Saba T. (2022). Skin cancer detection from dermoscopic images using deep learning and fuzzy k-means clustering. Microsc. Res. Techniq..

[75] Abo-Elnaga Y., Nasr S. (2022). K-means cluster interactive algorithm-based evolutionary approach for solving bilevel multi-objective programming problems. Alex. Eng. J..

[76] Rong H., Ramirez-Serrano A., Guan L., Gao Y. (2020). Image object extraction based on semantic detection and Improved K-Means algorithm. IEEE Access.

[77] Ovchinnikov V., Karplus M., Vanden-Eijnden E. (2011). A traveling-salesman based automated path searching method for functional conformational changes of biological macromolecules. J. Chem. Phys..

[78] Maragliano L., Vanden-Eijnden E., Roux B. (2009). Free energy and kinetics of conformational transitions from voronoi tessellated milestoning with restraining potentials. J. Chem. Theory Comput..

[79] Vanden-Eijnden E., Venturoli M. (2009). Supervised learning and the finite-temperature string method for computing committor functions and reaction rates. J. Chem. Phys..

[80] Xue C., Sun J., Song G.B., Kang B., Xia Y.X. (2008). Review on B→δ phase transition of HMX. Chin. J. Energ. Mater..

[81] Abreu J., Rico-Juan J. (2014). A new iterative algorithm for computing a quality approximate median of strings based on edit operations. Pattern Recognit. Lett..

[82] Henikoff J.G., Henikoff S. (1996). Blocks database and its applications. Method. Enzymol..

[83] Mirabal P., Abreu J., Seco D. (2019). Assessing the best edit in perturbation-based iterative refinement algorithms to compute the median string. Pattern Recognit. Lett..

[84] Maragliano L., Vanden-Eijnden E. (2007). On-the-fly string method for minimum free energy paths calculation. Chem. Phys. Lett..

[85] Norris J.R., Markov C. (2004). Cambridge Series in Statistical and Porbabilistic Mathematics.

[86] Rong C., Zhao D., Zhou T., Liu S., Yu D., Liu S. (2019). Homogeneous molecular systems are positively cooperative, but charged molecular systems are negatively cooperative. J. Phys. Chem. Lett..

[87] Ren F.D., Shi W.J., Cao D.L., Li Y.X., Liu L.L., Gao L. (2021). A theoretical investigation into the cooperativity effect on the TNT melting point under external electric field. J. Mol. Model..

[88] Elishav O., Podgaetsky R., Meikler O., Hirshberg B. (2023). Collective variables for conformational polymorphism in molecular crystals. J. Phys. Chem. Lett..

[89] Giberti F., Salvalaglio M., Mazzotti M., Parrinello M. (2015). Insight into the nucleation of urea crystals from the melt. Chem. Eng. Sci..

[90] Tribello G.A., Giberti F., Sosso G.C., Salvalaglio M., Parrinello M. (2017). Analyzing and driving cluster formation in atomistic simulations. J. Chem. Theory Comput..

[91] Gimondi I., Salvalaglio M. (2017). CO_2_ packing polymorphism under pressure: Mechanism and thermodynamics of the I-III polymorphic transition. J. Chem. Phys..

[92] Francia N.F., Price L.S., Nyman J., Price S.L., Salvalaglio M. (2020). Systematic finite-temperature reduction of crystal energy landscapes. Cryst. Growth. Des..

[93] Samanta A., Chen W.E.M., Yu T., Tuckerman M. (2014). Sampling saddle points on a free energy surface. J. Chem. Phys..

[94] Cuendet M.A., Tuckerman M.E. (2014). Free energy reconstruction from metadynamics or adiabatic free energy dynamics simulations. J. Chem. Theory Comput..

[95] Yu T.Q., Tuckerman M.E. (2011). Temperature-accelerated method for exploring polymorphism in molecular crystals based on free Energy. Phys. Rev. Lett..

[96] Piaggi P.M., Parrinello M. (2017). Entropy based fingerprint for local crystalline order. J. Chem. Phys..

[97] Piaggi P.M., Valsson O., Parrinello M. (2017). Enhancing entropy and enthalpy fluctuations to drive crystallization in atomistic simulations. Phys. Rev. Lett..

[98] Neha, Mondal S., Kumari N., Karmakar T. (2022). Collective variables for crystallization simulations-from early developments to recent advances. ACS Omega.

[99] Turnbull D., Fisher J.C. (1949). Rate of Nucleation in Condensed Systems. J. Chem. Phys..

[100] Rice B.M., Sahu S., Owens F.J. (2002). Density functional calculations of bond dissociation energies for NO_2_ scission in some nitroaromatic molecules. J. Mol. Struct. Theochem..

[101] Aina A.A., Misquitta A.J., Phipps M.J.S., Price S.L. (2019). Charge Distributions of Nitro Groups within Organic Explosive Crystals: Effects on Sensitivity and Modeling. ACS Omega.

[102] Politzer P., Murray J.S. (2014). Impact sensitivity and crystal lattice compressibility/free space. J. Mol. Model..

[103] Michalchuk A.A.L., Trestman M., Rudić S., Portius P., Fincham P.T., Pulham C.R., Morrison C.A. (2019). Predicting the reactivity of energetic materials: An ab initio multi-phonon approach. J. Mater. Chem. A.

[104] Atceken N., Hemingway J., Bull C.L., Liu X.J., Michalchuk A.A.L., Konar S., Morrison C.A., Pulham C.R. (2023). High-pressure structural studies and pressure-induced sensitisation of 3,4,5-trinitro-1H-pyrazole. Phys. Chem. Chem. Phys..

[105] Christopher I.L., Pulham C.R., Michalchuk A.A.L., Morrison C.A. (2023). Is the impact sensitivity of RDX polymorph dependent?. J. Chem. Phys..

[106] Michalchuk A.A.L., Hemingway J., Morrison C.A. (2021). Predicting the impact sensitivities of energetic materials through zone-center phonon up-pumping. J. Chem. Phys..

[107] Michalchuk A.A.L., Rudić S., Pulham C.R., Morrison C.A. (2021). Predicting the impact sensitivity of a polymorphic high explosive: The curious case of FOX-7. Chem. Commun..

[108] Hunter S., Sutinen T., Parker S.F., Morrison C.A., Williamson D.M., Thompson S., Gould P.J., Pulham C.R. (2013). Experimental and DFT-D Studies of the Molecular Organic Energetic Material RDX. J. Phys. Chem. C.

[109] Jolliffe I. (2002). Principal Component Analysis.

[110] http://www.ks.uiuc.edu/Research/namd/.

[111] MacKerell A., Wiorkiewicz-Kuczera J., Karplus M. (1995). An all-atom empirical energy function for the simulation of nucleic acids. J. Am. Chem. Soc..

[112] MacKerell A., Bashford D., Bellott M., Dunbrack R., Evanseck J., Field M., Fischer S., Gao J., Guo H., Ha S. (1998). All-atom empirical potential for molecular modeling and dynamics studies of proteins. J. Phys. Chem. B.

[113] Phillips J., Braun R., Wang W., Gumbart J., Tajkhorshid E., Villa E., Chipot C., Skeel R., Kale L., Schulten K. (2005). Sire: An interoperability engine for prototyping algorithms and exchanging information between molecular simulation programs. J. Comput. Chem..

[114] Darden T., York D., Pedersen L. (1993). Particle mesh ewald: An N⋅log(N) method for ewald sums in large systems. J. Chem. Phys..

