# Supplementary material for: Theoretical Investigation into Polymorphic Transformation between β-HMX and δ-HMX by Finite Temperature String
Source: Molecules. 2024 Oct 11;29(20):4819. doi: 10.3390/molecules29204819 (PMC11510520; doi:10.3390/molecules29204819)
Supplement: Supplementary file 1 [file molecules-29-04819-s001.zip › molecules-3218023-supplementary.pdf]

Supporting Information

# Theoretical investigation into polymorphic transformation between *β*-HMX and *δ*-HMX by finite temperature string

Xiu-mei Jia, Zhen-dong Xin, Yi-zheng Fu Hong-ji Duan

**Table S1** Lattice parameters of *β*-HMX and *δ*-HMX

**Table S2** Peaks in the pair distribution function for the reference structures of the *β*-HMX and *δ*-HMX crystals at 480 K with a cutoff of 10.0 Å.

**Table S3** Average peak locations and concentrations parameters for the *β*-HMX and *δ*-HMX crystals at 480 K with a cutoff of 10.0 Å.

**Table S4** Average peak locations for the replicas by average-based sampling at 480 K.

**Table S5** Values of the partition coefficient (PC) and partition entropy (PE).

**Table S6** Average peak locations and concentrations parameters for the β-HMX and δ-HMX crystals at 510 K, 450 K and 420 K.

**Table S1** Lattice parameters of *β*-HMX and *δ*-HMX [20]

| | **Space group** | ***a*(Å)** | ***b*(Å)** | ***c*(Å)** | ***β*(°)** | ***Z*** | ***ρ*(g/cm³)** |
|---|---|---|---|---|---|---|---|
| *β*-HMX | $P2_1/c$ | 6.54 | 11.05 | 8.70 | 124.4 | 2 | 1.905 |
| *δ*-HMX | $P6_1$ | 7.71 | 7.71 | 32.55 | 90.0 | 6 | 1.80 |

**Table S2** Peaks in the pair distribution function for the reference structures of the $\beta$-HMX and $\delta$-HMX crystals at 480 K with a cutoff of 10.0 Å.

| | ***β*-HMX** | |
| --- | --- | --- |
| ***r*(Å)** | **$\phi_{\hat{r}}$ (°)** | **$\phi_q$ (°)** |
| 6.62 | 53.18 | 32.18 |
| 7.51 | 27.01 | 57.22 |
| 7.88 | 62.37 | 65.18 |
| | ***δ*-HMX** | |
| 6.50 | 12.51 | 10.6 |
| 8.92 | 67.36 | 157.63 |
| 9.73 | 86.53 | 133.19 |

**Table S3** Average peak locations and concentrations parameters for the *β*-HMX and *δ*-HMX crystals at 480 K with a cutoff of 10.0 Å.

| | | ***β*-HMX** | | | |
|---|---|---|---|---|---|
| ***r*(Å)** | **1/*σ*² (Å⁻¹)** | $\phi_{\hat{r}}$ **(°)** | $\eta_{\hat{r}}^{\alpha}$ | $\phi_{q}$ **(°)** | $\eta_{q}^{\alpha}$ |
| 6.58 | 28.43 | 56.37 | 15.22 | 33.91 | 23.17 |
| 7.53 | 21.68 | 32.15 | 11.18 | 49.18 | 12.66 |
| 7.91 | 46.24 | 59.01 | 9.38 | 68.23 | 19.28 |
| | | ***δ*-HMX** | | | |
| 6.56 | 26.82 | 16.18 | 16.28 | 15.17 | 18.13 |
| 8.91 | 15.79 | 62.17 | 26.15 | 150.28 | 12.71 |
| 9.68 | 54.63 | 88.15 | 19.85 | 135.38 | 25.63 |

**Table S4** Average peak locations for the replicas by average-based sampling at 480 K.

| | **1#** | | | **2#** | | | **3#** | | | **4#** | | |
|---|---|---|---|---|---|---|---|---|---|---|---|---|
| $r$(Å) | 6.58 | 7.53 | 7.91 | 6.59 | 7.54 | 7.91 | 6.57 | 7.55 | 7.91 | 6.59 | 7.56 | 7.91 |
| $1/\sigma^2$(Å$^{-1}$) | 28.43 | 21.68 | 46.24 | 55.18 | 22.65 | 16.18 | 19.50 | 17.51 | 28.22 | 27.13 | 24.02 | 31.68 |
| | **5#** | | | **6#** | | | **7#** | | | **8#** | | |
| $r$(Å) | 6.58 | 7.57 | 7.91 | 6.60 | 7.60 | 7.93 | 6.56 | 7.63 | 7.95 | 6.59 | 7.78 | 7.96 |
| $1/\sigma^2$(Å$^{-1}$) | 22.13 | 17.51 | 29.08 | 30.56 | 32.68 | 21.23 | 26.53 | 36.18 | 29.13 | 32.17 | 23.18 | 26.92 |
| | **9#** | | | **10#** | | | **11#** | | | **12#** | | |
| $r$(Å) | 6.56 | 7.82 | 7.99 | 6.55 | 7.81 | 8.02 | 6.57 | 8.83 | 8.25 | 6.55 | 8.85 | 8.40 |
| $1/\sigma^2$(Å$^{-1}$) | 23.98 | 25.18 | 27.11 | 13.78 | 22.40 | 11.37 | 25.39 | 35.69 | 22.18 | 29.16 | 32.40 | 39.26 |
| | **13#** | | | **14#** | | | **15#** | | | **16#** | | |
| $r$(Å) | 6.58 | 8.86 | 8.62 | 6.57 | 8.87 | 8.92 | 6.56 | 8.90 | 9.08 | 6.55 | 8.89 | 9.23 |
| $1/\sigma^2$(Å$^{-1}$) | 37.38 | 22.78 | 29.18 | 42.05 | 26.03 | 10.67 | 19.52 | 51.98 | 32.06 | 32.58 | 57.13 | 17.62 |
| | **17#** | | | **18#** | | | **19#** | | | **20#** | | |
| $r$(Å) | 6.58 | 8.91 | 9.46 | 6.55 | 8.92 | 9.69 | 6.57 | 8.92 | 9.65 | 6.56 | 8.91 | 9.68 |
| $1/\sigma^2$(Å$^{-1}$) | 19.56 | 28.67 | 26.53 | 12.31 | 42.28 | 26.23 | 45.18 | 33.21 | 39.17 | 26.82 | 15.79 | 54.63 |

**Table S5** Values of the partition coefficient (PC) and partition entropy (PE).

| | **1#** | **2#** | **3#** | **4#** | **5#** | **6#** | **7#** | **8#** | **9#** | **10#** |
|---|---|---|---|---|---|---|---|---|---|---|
| PC | 0.79 | 0.81 | 0.76 | 0.76 | 0.82 | 0.53 | 0.83 | 0.81 | 0.81 | 0.76 |
| PE | 0.28 | 0.52 | 0.33 | 0.31 | 0.42 | 0.40 | 0.38 | 0.39 | 0.50 | 0.39 |
| | **11#** | **12#** | **13#** | **14#** | **15#** | **16#** | **17#** | **18#** | **19#** | **20#** |
| PC | 0.65 | 0.82 | 0.87 | 0.73 | 0.81 | 0.83 | 0.76 | 0.82 | 0.80 | 0.65 |
| PE | 0.28 | 0.43 | 0.55 | 0.50 | 0.35 | 0.32 | 0.28 | 0.45 | 0.51 | 0.29 |

**Table S6** Average peak locations and concentrations parameters for the *β*-HMX and *δ*-HMX crystals at 510 K (in smooth), 450 K (in bold) and 420 K (in italic) with a cutoff of 10.0 Å.

| | | ***β*-HMX** | | | |
|---|---|---|---|---|---|
| ***r*(Å)** | **1/$\sigma^2$ (Å$^{-1}$)** | $\phi_{\hat{r}}$ **(°)** | $\eta_{\hat{r}}^{\alpha}$ | $\phi_q$ **(°)** | $\eta_q^{\alpha}$ |
| 6.56 **6.52** | 27.52 **28.79** | 58.22 **69.18** | 16.83 **18.19** | 30.84 **35.68** | 21.25 **25.18** |
| *6.57* | *26.18* | *67.25* | *26.13* | *30.63* | *22.30* |
| 7.52 **7.58** | 22.33 **20.12** | 31.66 **38.27** | 19.03 **10.35** | 55.28 **47.62** | 15.27 **16.27** |
| *7.51* | *17.96* | *39.26* | *5.37* | *50.19* | *17.27* |
| 7.92 **7.86** | 43.55 **42.18** | 67.89 **52.18** | 18.22 **11.17** | 66.18 **87.27** | 21.15 **13.05** |
| *7.90* | *45.03* | *51.76* | *5.32* | *61.17* | *17.65* |
| | | ***δ*-HMX** | | | |
| 6.55 **6.57** | 29.11 **27.10** | 22.18 **11.03** | 19.17 **16.52** | 19.25 **11.63** | 15.09 **24.16** |
| *6.55* | *29.15* | *10.52* | *11.10* | *18.49* | *17.23* |
| 8.93 **8.94** | 16.40 **18.13** | 53.66 **63.86** | 15.32 **23.27** | 113.25 **111.33** | 15.43 **13.16** |
| *8.91* | *17.63* | *67.19* | *29.19* | *125.89* | *18.03* |
| 9.67 **9.69** | 58.19 **50.33** | 80.17 **80.28** | 21.63 **17.92** | 132.02 **128.19** | 28.55 **20.32** |
| *9.66* | *50.17* | *82.95* | *11.63* | *116.67* | *26.71* |
